# Device runtime and costs of cardiac resynchronization therapy pacemakers – a health claims data analysis

**DOI:** 10.3205/000304

**Published:** 2022-03-04

**Authors:** Moritz Hadwiger, Nikolaos Dagres, Gerhard Hindricks, Helmut L’hoest, Ursula Marschall, Alexander Katalinic, Fabian-Simon Frielitz

**Affiliations:** 1 Institute of Social Medicine and Epidemiology, University of Lübeck, Germany; 2 Heart Center Leipzig, University of Leipzig, Germany; 3 BARMER, Wuppertal, Germany

**Keywords:** cardiac resynchronization therapy, device longevity, device runtime, battery runtime, health claims data

## Abstract

**Introduction:** This study investigates the runtime and costs of biventricular defibrillators (CRT-D) and biventricular pacemakers (CRT-P). Accurate estimates of cardiac resynchronization therapy (CRT) device runtime across all manufactures are rare, especially for CRT-P.

**Methods:** Health claims data of a large nationwide German health insurance was used to analyze CRT device runtime. We defined device runtime as the time between the date of implantation and the date of generator change or removal. The median costs for implantation, change, and removal of a CRT device were calculated accordingly.

**Results:** In total, the data set comprises 17,826 patients. A total of 4,296 complete runtimes for CRT-D devices and 429 complete runtimes for CRT-P devices were observed. Median device runtime was 6.04 years for CRT-D devices and 8.16 years for CRT-P devices (log-rank test p<0.0001). The median cost of implantation for a CRT-D device was 14,270 EUR, and for a CRT-P device 9,349 EUR.

**Conclusions:** Compared to CRT-P devices, CRT-D devices had a significantly shorter device runtime of about two years. Moreover, CRT-D devices were associated with higher cost. The study provides important findings that can be utilized by cost-effectiveness analyses.

## Zusammenfassung

**Einleitung:** Diese Studie untersucht die Laufzeit und Kosten von biventrikulären Defibrillatoren (CRT-D) und biventrikulären Schrittmachern (CRT-P). Genaue Schätzungen der Laufzeit von Geräten für die kardiale Resynchronisationstherapie (CRT) über alle Hersteller sind selten, insbesondere für CRT-P.

**Methoden:** Zur Analyse der CRT-Gerätelaufzeit wurden Routinedaten einer großen bundesweiten deutschen Krankenkasse verwendet. Wir definierten die Gerätelaufzeit als die Zeit zwischen dem Datum der Implantation und dem Datum des Generatorwechsels oder der Entfernung. Die medianen Kosten für Implantation, Wechsel und Entfernung eines CRT-Gerätes wurden ebenfalls berechnet.

**Ergebnisse:** Insgesamt umfasst der Datensatz 17.826 Patienten. Es wurden insgesamt 4.296 komplette Laufzeiten für CRT-D-Geräte und 429 komplette Laufzeiten für CRT-P-Geräte beobachtet. Die mediane Geräte-Laufzeit betrug 6,04 Jahre für CRT-D-Geräte und 8,16 Jahre für CRT-P-Geräte (Log-Rank-Test p<0,0001). Die medianen Implantationskosten betrugen 14.270 EUR für ein CRT-D-Gerät und 9.349 EUR für ein CRT-P-Gerät.

**Fazit:** Im Vergleich zu CRT-P-Geräten hatten CRT-D-Geräte eine signifikant kürzere Gerätelaufzeit von etwa zwei Jahren. Außerdem waren CRT-D-Geräte mit höheren Kosten verbunden. Die Studie liefert wichtige Ergebnisse, die in Kosten-Effektivitätsanalysen verwendet werden können.

## 1 Introduction

Cardiac resynchronization therapy (CRT) is an established form of treatment for heart failure patients. In addition to the survival benefit, the runtime of a CRT device and its cost are decisive factors in evaluating the cost-effectiveness of CRT devices. Like any surgery, CRT device replacement carries the risk of complications such as infections [1], [2] and puts an economic burden on the healthcare system.

Biventricular defibrillator (CRT-D) or biventricular pacemaker (CRT-P) [3] implantation is indicated for primary prevention in patients with symptomatic heart failure of New York Heart Association (NYHA) classes II–IV, reduced ejection fraction ≤35%, and broad QRS complex. A CRT-D device combines the functions of a CRT-P device with the addition of a defibrillator [4]. Due to shocks delivered by the defibrillator in the CRT-D device, the battery may be depleted earlier.

In health economic modelling approaches, a high variability of the assumed CRT device runtime could be observed [5]. However, CRT device runtime is one of the most important input parameters for evaluating cost-effectiveness, since CRT-D devices are more expensive than CRT-P devices and the costs of device replacement and device runtime are interrelated. In addition, a modeling study found that longer battery runtime reduces adverse events such as infections, device revisions, and costs [6].

Several clinical studies report the device longevity of CRT-D or implantable cardioverter defibrillators [7], [8], [9], and recent medical publications focus more on runtime differences between device manufacturers [10]. However, to our knowledge, information on CRT-P longevity is scarce [11], yet for cost-effectiveness analysis, valid device runtime estimates are crucial. In addition, device longevity is often defined as the time until battery depletion [8], [9]. However, from a public health perspective, the overall median runtime is relevant regardless of the manufacturer. Therefore, this study aims to evaluate the runtime and costs of CRT devices from implantation to replacement for any reason, using health claims data of a major German statutory health insurance provider for the years 2006 to 2019.

## 2 Methods

### 2.1 Data source and setting

To evaluate device runtime and to estimate costs, a retrospective health claims data analysis was performed. The analysis was based on health claims data from BARMER, a large nationwide statutory health insurance with 8.8 million insured [12]. In Germany, health insurance is mandatory, and the vast majority (approx. 90%) of the German population is insured in a statutory health insurance such as BARMER [13]. The BARMER database contains the complete longitudinal anonymized health claims data of all insured persons for the years 2005 to 2019 with information on e.g. costs, utilization, and socio-demographics. The age and gender distribution of BARMER insured can be generalized to the German population [14], [15].

### 2.2 Sample selection

To determine the runtime of CRT devices, all CRT implantations or device upgrades in the database were selected from the years 2006–2019. Implantation, change, and removal of a device were selected according to the Operation and Procedure Codes (OPS), an adaptation of the International Classification of Procedures in Medicine [16]. The first year (2005) was excluded from the analysis because OPS codes could not be distinguished between CRT-P and CRT-D devices.

**Figure 1:**
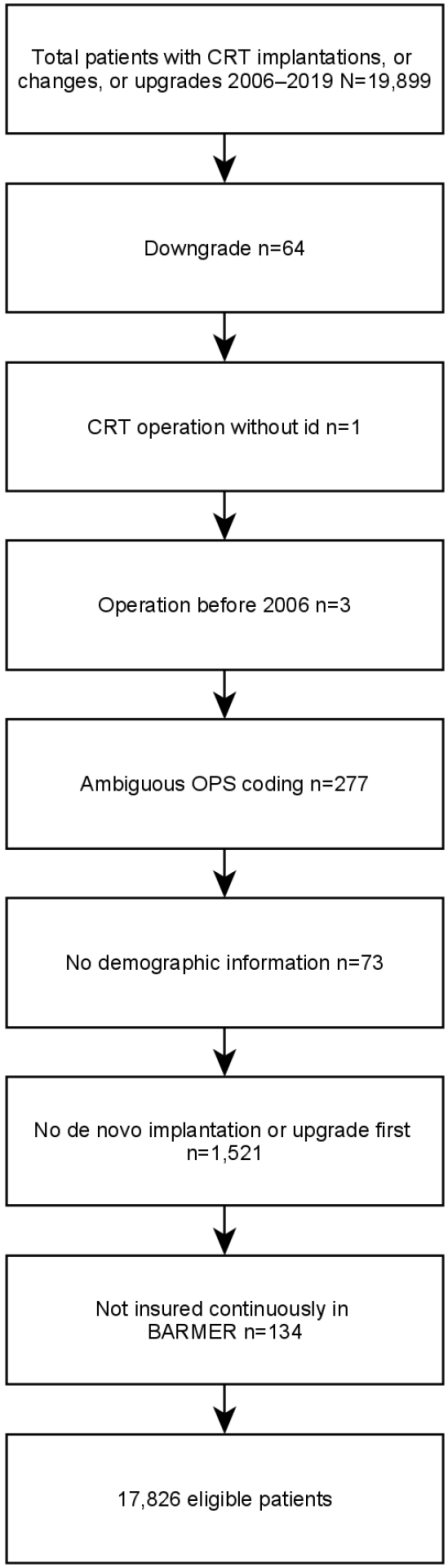
Flowchart

All patients in the database who were coded with a CRT device implantation, device change, or device upgrade were selected (N=19,899). Patients who had been downgraded during the observational period were excluded (n=64), as well as patients who had an ambiguous OPS coding (n=277). Patients in whom neither de novo implantation nor device upgrade was coded first were excluded for analysis (n=1,521), as were patients who were not continuously insured in BARMER after CRT implantation (n=134). The final sample included 17,826 patients. More detailed information on the exclusion of patients is given in the flowchart (Figure 1 (Fig. 1)).

### 2.3 Outcomes

The main outcome was device runtime. The index date was defined as the date of device implantation or upgrade to a CRT device. Follow-up was defined as the time between the implantation and the date of generator change or removal for any reason or censoring. Reasons for censoring included death of the patient, a change in health insurer, or that the device was still in use at the end of the observation period, i.e. Dec. 31, 2019. Beside device runtime, considered outcomes were median costs in euros for implantation, generator change, and removal per device. To account for inflation in prices, only cost data for 2019 were used. In addition, we illustrate the median price development over time per device.

### 2.4 Statistical analysis

Median runtime per device type was calculated (two-sided 95% confidence interval). Time-to-event rates for device replacement were illustrated by Kaplan-Meier curves. Differences between CRT-D and CRT-P were analyzed using the long-rank test. A *p*-value of <0.05 was considered statistically significant. Statistical analysis was carried out in “R” [17]. In order to check if results were robust, we performed three sensitivity analyses.

## 3 Results

**Figure 2:**
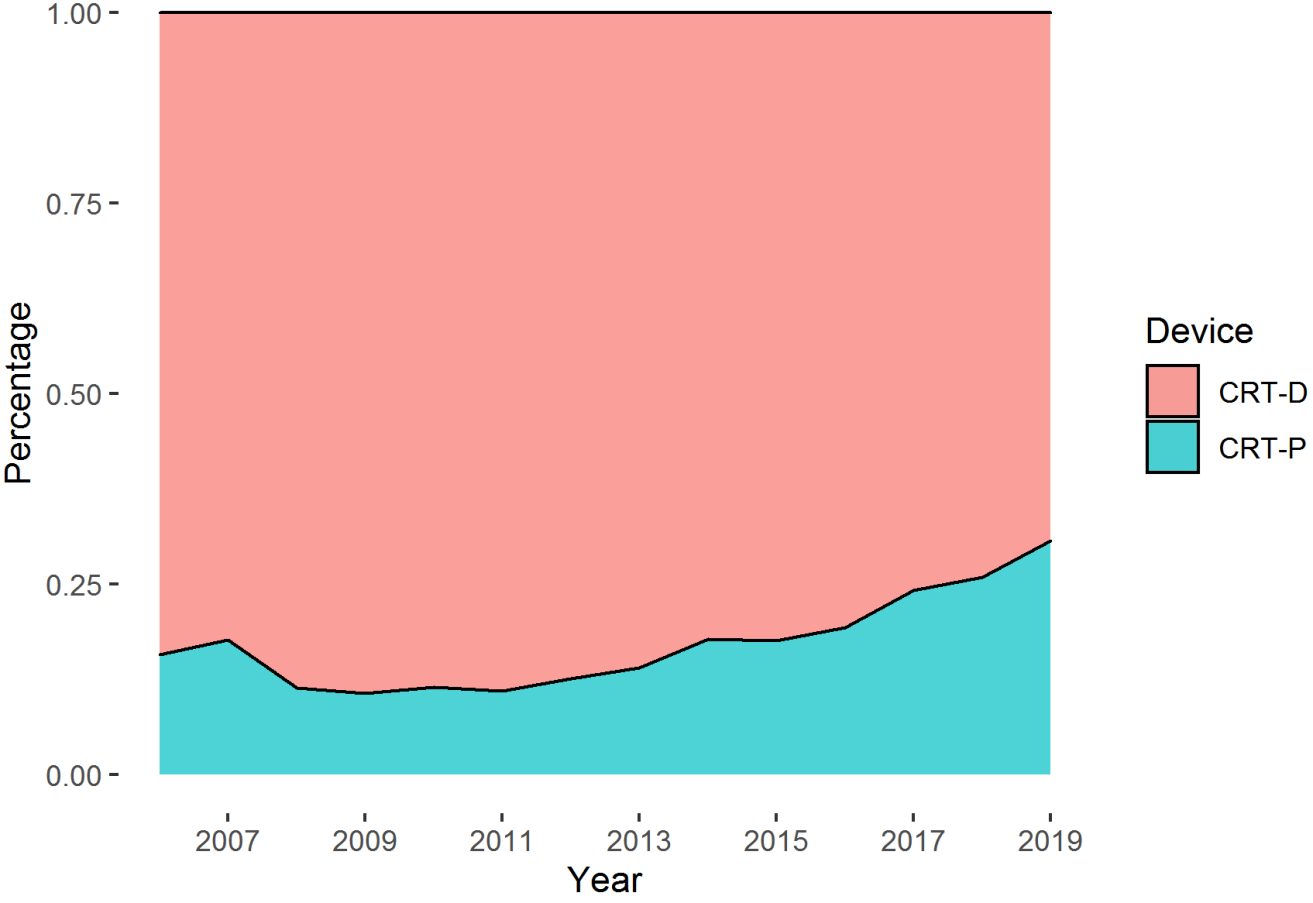
Proportion of CRT-D and CRT-P implantations per year out of all CRT implantations

In total, 17,826 patients had a CRT implantation or upgrade in the years from 2006 to 2019. These patients caused 18,246 device implantations or upgrades, 4,043 generator changes, and 371 device removals. Of these 22,660 cases, 18,404 were CRT-D devices and 4,256 were CRT-P devices. Figure 2 (Fig. 2) illustrates the annual proportion of CRT devices types out of all CRT implantations per year. CRT-P cases were comparatively low, but have risen slightly in recent years. Per patient, the median follow-up time was 2.89 years (interquartile range 1.26–4.79 years). On average, CRT-P patients were 6.4 years older than CRT-D patients at their first CRT implantation, and more often female (Table 1 (Tab. 1)).

**Figure 3:**
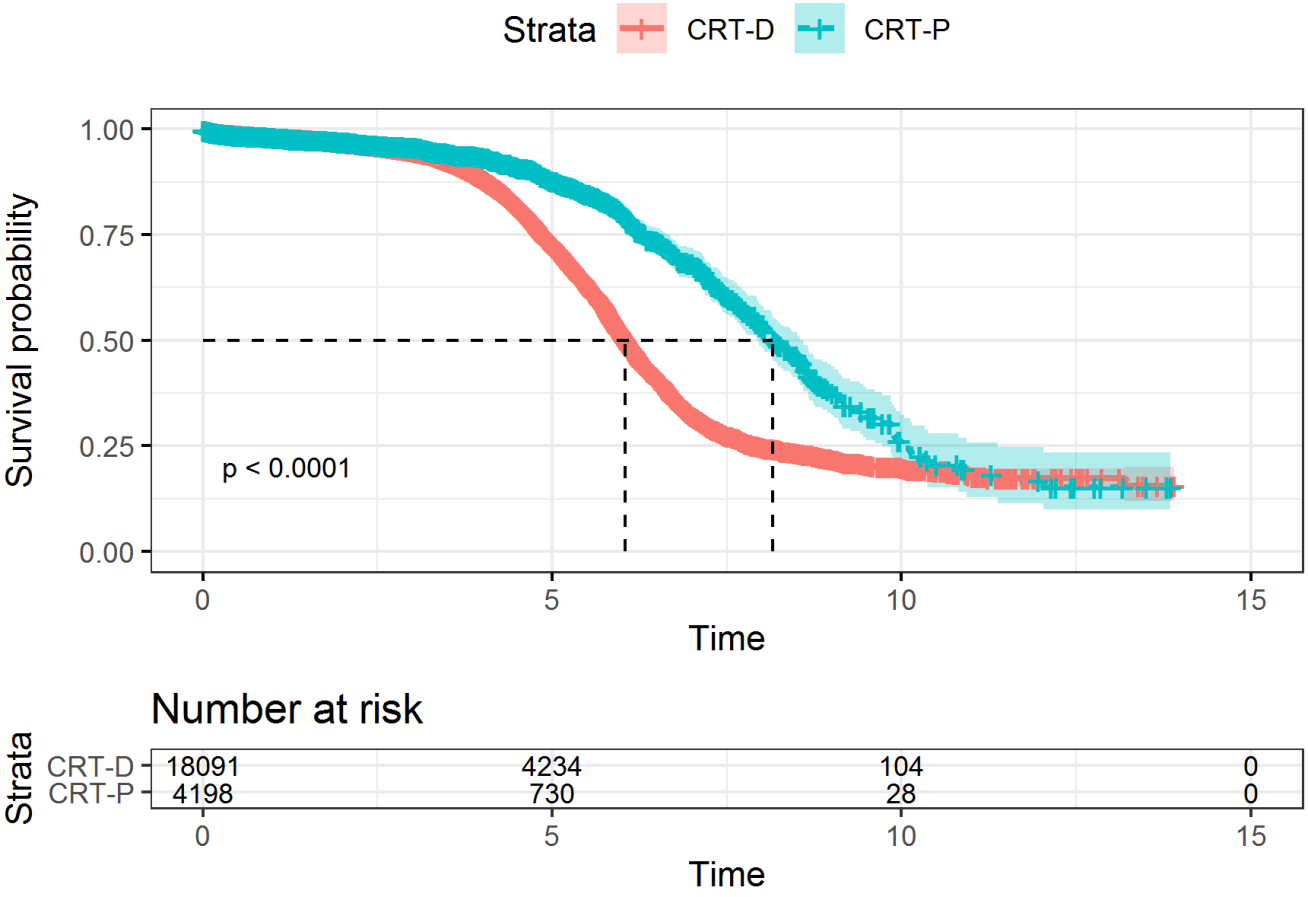
Kaplan-Meier curves for event-free survival of CRT devices (time to generator change/removal): CRT-D (blue), CRT-P (red)

A total of 4,725 complete runtimes were observed. Of these, 4,296 were CRT-D runtimes and 429 were CRT-P runtimes. The median device runtime was 6.04 years (95% confidence interval (CI) 6.00; 6.10) for CRT-D devices and 8.16 years (95% CI 7.93; 8.59) for CRT-P devices. The device survival is depicted in the Kaplan-Meier curves (Figure 3 (Fig. 3)), indicating a difference in device runtime per type (log-rank test *p*<0.0001).

To validate the results of CRT runtimes, we conducted three sensitivity analyses. In the first analysis, only cases from 2010 or later were selected, which resulted in a median runtime of 6.29 years (95% CI 6.22; 6.39) for CRT-D and 8.00 years (95% CI 7.65; 8.44) for CRT-P. Second, observations with a runtime longer than 10 years were excluded, yielding a median runtime of 6.01 years (95% CI 5.95; 6.06) for CRT-D devices and 7.96 years (95% CI 7.72; 8.27) for CRT-P devices. In the third sensitivity analysis, just one runtime observation per patient was considered, resulting in a median runtime of 6.08 years (95% CI 6.02; 6.15) for CRT-D devices and 8.27 years (95% CI 8.00; 8.66) for CRT-P devices.

The median cost of a CRT-D device implantation in 2019 was 14,270 EUR, 8,417 EUR for a generator change, and 12,258 EUR for a device removal. The median cost was 9,349 EUR for a CRT-P implantation, 5,226 EUR for a generator change, and 13,019 EUR for a device removal. Further information is given in Table 1 (Tab. 1).

**Figure 4:**
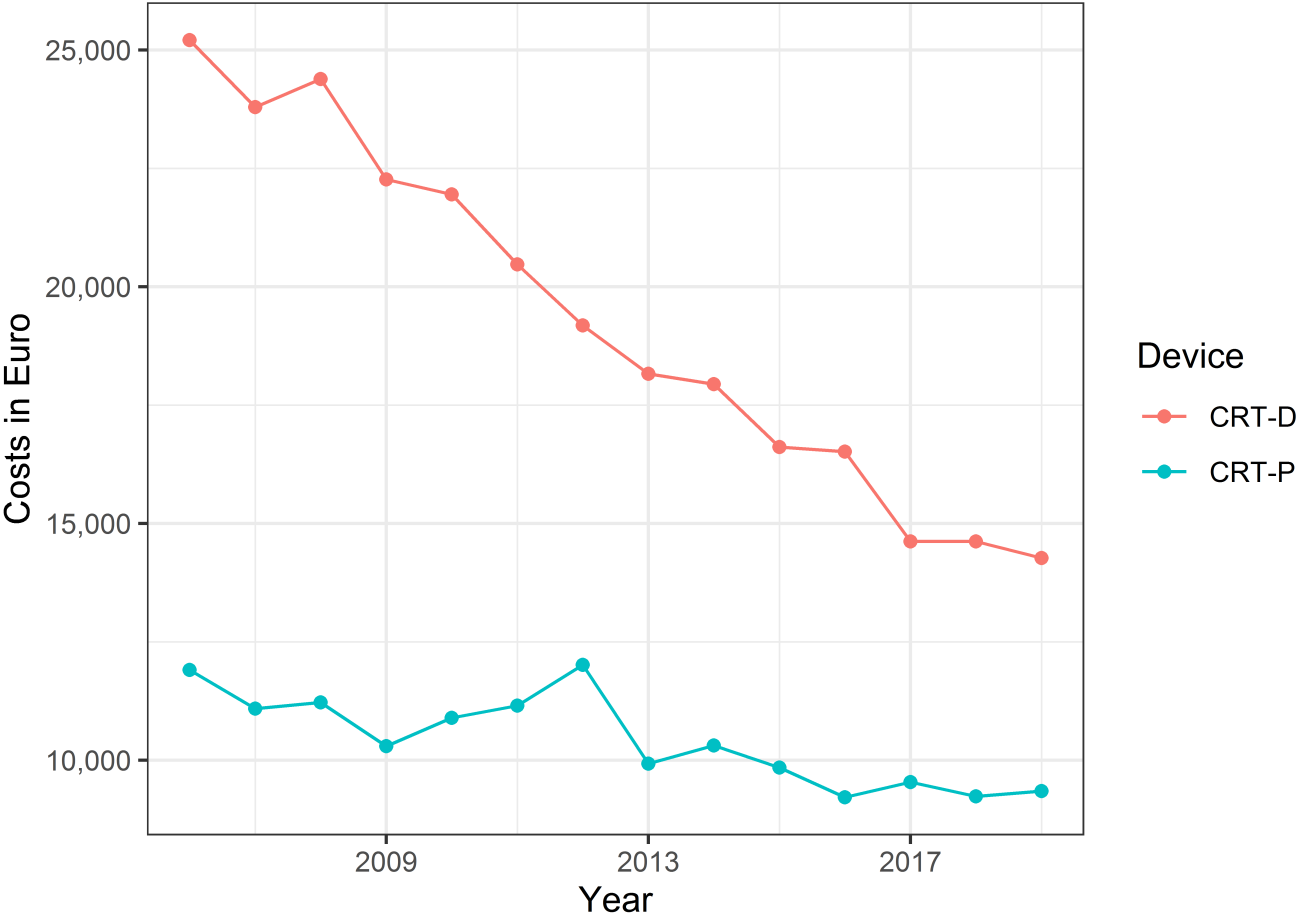
Development of the median implantation cost of the CRT devices

In Figure 4 (Fig. 4), the development of the median implantation cost of the CRT devices over time is depicted. Cost for CRT-P devices was stable over time, whereas cost for a CRT-D implantation decreased constantly.

## 4 Discussion

The analysis of CRT device runtime with routine data of a major German health insurance provider resulted in a median device runtime of 6.04 years for CRT-D and 8.16 years for CRT-P. CRT-D devices had a significantly (*p*<0.0001) shorter runtime compared to CRT-P devices. The results are robust in three sensitivity analyses. CRT-D operations were associated with higher costs for implantation and generator change. Costs for device removal were slightly higher in CRT-P devices.

Overall, the result of the CRT-D runtime is similar to most other estimates from related studies. Landolina et al. [8] report a probability of survival free from battery depletion of 54% at 5 years for CRT-D. Zanon et al. [9] state a median device longevity of 4.9 (4.0–5.7) years for CRT-D. Time to battery depletion also differs by manufacturer and deviates significantly from published product performance reports [8], [18], [19]. In our analysis, we used survival data, and devices that were still operating at the end of the observation period had to be censored, which could potentially reduce the overall runtime.

The median longevity estimates reported in a final appraisal of a manufacturer’s submission from the National Institute for Health and Care Excellence is the only source to our knowledge which reports the longevity of CRT-P devices. The median survival time of CRT devices is given as 10.4 years for CRT-P and 5.8 years for CRT-D. Estimates were calculated using Weibull curves. The runtime was calculated using data from the NHS Central Cardiac Audit Database for the years 2000 to 2011 [11]. The longevity result for CRT-D devices is comparable. However, the median runtime of CRT-P devices differs considerably from our results. The stated median longevity of CRT-P devices is 2.24 years higher than our results. Colquitt et al. [4] point out that clinicians have informed them that these runtimes may be overestimated.

Precise longevity estimates of CRT devices are important for health economic evaluations, as it is known that device runtime is a critical parameter in assessing cost-effectiveness [5]. Moreover, device cost is an important input parameter which is interconnected with device longevity. If the device lasts longer, the device must not be replaced as often, resulting in cost reduction [20]. The assumed device longevity in cost-effectiveness analysis differs approximately ±2 years from the estimated result for CRT-P devices [4], [21], [22], [23], [24].

From the patient’s perspective, a long runtime of the device is desirable because each admission to a hospital is associated with the risk of infection, and the operation with the risk of complications. Furthermore, CRT-D devices are more likely to cause problems compared to CRT-P devices [2], which could also be a reason for the shorter device runtime. A device runtime that matches the life expectancy of patients would save additional hospital admissions [25]. In addition, older age is positively correlated with complications in CRT devices [26].

Costs for CRT-D therapy are considerably higher than for CRT-P therapy. On average, the cost difference between a CRT-D implantation and a CRT-P implantation is 4,921 EUR, which is nearly half of the cost of a CRT-P device. Device cost is an important parameter in cost-effectiveness analysis, which is interconnected with the device runtime, and costs for device change are 3,191 EUR higher for CRT-D devices. It is known that device runtime is a critical parameter in assessing cost-effectiveness [5]. If the device lasts longer, the device must not be replaced as often, resulting in cost reduction [20]. Device costs decreased over time, especially the implantation costs of CRT-D devices (Figure 4 (Fig. 4)), which may be due to changes in the relative cost weights in the German diagnosis-related group codes. Holding all other parameters constant, the reduced cost difference has a positive effect on the incremental cost-benefit ratio of CRT-D devices compared to CRT-P devices. There is no randomized clinical trial with sufficient power for a direct comparison between CRT-P and CRT-D, and the additional defibrillator is still controversial. The still substantial cost differences between the two devices highlight the importance of cost-effectiveness analysis.

Due to the data’s special characteristics, the analysis is limited in several ways. In the health claims data, the manufacturer of the device or the battery type could not be observed. For this reason, it was not possible to examine differences in the runtime of devices between manufacturers, which may vary significantly [9], [27]. Second, due to data unavailability, no influencing control variables like pacing mode could be included in the analysis. Third, a complete cost-effectiveness analysis would require survival data from CRT patients. However, for a comprehensive evaluation of the two CRT devices, this analysis provides robust estimates of device runtime, implantation costs, and change costs.

The usage of health claims data has major strengths. Real-world data with a long timeframe were used for the estimation of the device runtime and device complications. The analysis did not examine the battery longevity, but the entire device runtime including replacement due to device malfunctions, among other reasons, which is the more relevant parameter for patients, clinicians, and health insurance providers. For the medical practice it is important to know how long a complete CRT device runtime is and not only the battery longevity.

## 5 Conclusions

Using health claims data of 17,826 patients for researching CRT device runtime revealed a median device runtime of 6.04 years for CRT-D and 8.16 years for CRT-P. Results were robust in various sensitivity analyses. Besides a shorter device runtime, the costs are higher in CRT-D devices. For clinical practice, estimates of device runtime provide additional information for device selection in patients who have an indication for a CRT-D or CRT-P device. In addition, proper runtime data are crucial for a reliable evaluation of cost-effectiveness in this patient group. More information on device longevity of CRT-P devices, data on the respective manufacturer, battery technology used, and reason for replacement are needed. The study provides important findings that can be utilized by cost-effectiveness analyses.

## Notes

### Availability of data

The data that support the findings of this study are owned by BARMER (Wuppertal, Germany) and are not publicly available.

### Funding

The study was funded by the German Federal Joint Committee (the highest decision-making body of the joint self-government of physicians, dentists, hospitals, and health insurance funds in Germany). Funding code: 01VSF17050.

### Competing interests

The authors declare that they have no competing interests.

## Figures and Tables

**Table 1:**
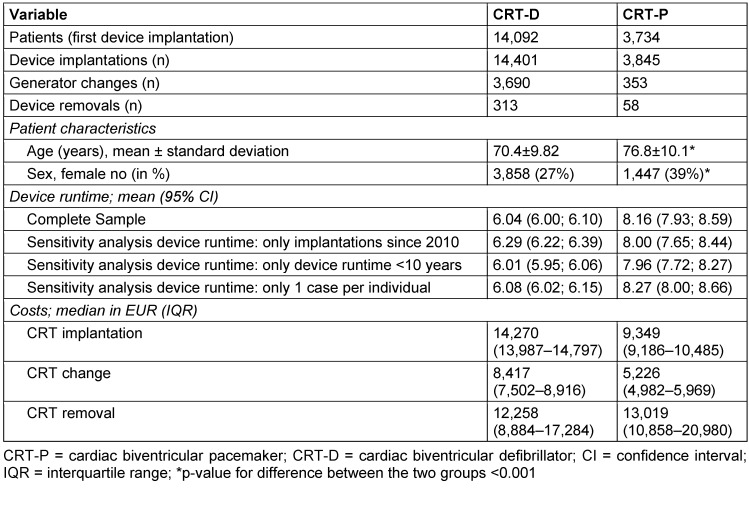
Patient characteristics, device runtime, and cost
